# Overall survival and cancer-specific survival were improved in local treatment of metastatic prostate cancer

**DOI:** 10.3389/fonc.2023.1130680

**Published:** 2023-05-03

**Authors:** Qi Miao, Zhihao Wei, Chenchen Liu, Yuzhong Ye, Gong Cheng, Zhengshuai Song, Kailei Chen, Yunxuan Zhang, Jiawei Chen, Changjie Yue, Hailong Ruan, Xiaoping Zhang

**Affiliations:** ^1^ Department of Urology, Union Hospital, Tongji Medical College, Huazhong University of Science and Technology, Wuhan, China; ^2^ Institute of Urology, Union Hospital, Tongji Medical College, Huazhong University of Science and Technology, Wuhan, China; ^3^ Department of Urology, The Central Hospital of Wuhan, Tongji Medical College, Huazhong University of Science and Technology, Wuhan, China

**Keywords:** metastatic prostate cancer, radical prostatectomy, external beam radiation therapy, radiotherapy, cancer-specific survival

## Abstract

**Background:**

For metastatic prostate cancer (mPCa), radical prostatectomy (RP) and radiation therapy (RT) may improve overall survival (OS) and cancer-specific survival (CSS). Compared with RT, RP shows significant advantages in improving patient outcomes. External beam radiation therapy (EBRT) even slightly elevates CSM with no statistical difference in OS compared with no local treatment (NLT).

**Objective:**

To evaluate OS and CSS after local treatment (LT) (including RP and RT) versus NLT in mPCa.

**Design, setting, and participants**

Within the Surveillance, Epidemiology and End Results (SEER) database (2000-2018), 20098 patients with metastatic prostate cancer were selected in this study, of which 19433 patients had no local treatment, 377 patients with radical prostate treatment, and 288 patients with RT.

**Outcome measurements and statistical analysis:**

Multivariable competing risks regression analysis after propensity score matching (PSM) was used to calculate CSM. Multivariable Cox regression analysis was used to identify the risk factors. Kaplan-Meier methods were used to calculate OS.

**Results and limitations:**

A total of 20098 patients were included: NLT (n = 19433), RP (n=377) and RT (n=288). In a competing risk regression analysis after PSM (ratio 1:1), RP resulted in a significantly lower CSM (hazard ratio [HR] 0.36, 95% confidence interval [CI] 0.29-0.45) than NLT, while RT showed a slightly lower CSM (HR 0.77, 95% CI 0.63-0.95). In a competing risk regression analysis after PSM (ratio 1:1), RP led to a lower CSM (HR 0.56, 95% CI 0.41-0.76) versus RT. As for all-cause mortality (ACM), RP (HR 0.37, 95% CI 0.31-0.45) and RT (HR 0.66, 95% CI 0.56-0.79). also showed a downward trend. In terms of OS, RP and RT significantly improved the survival probability compared with NLT, with the effect of RP being more pronounced. Obviously, older age, Gleason scores ≥8, AJCC T3-T4 stage, AJCC N1, AJCC M1b-M1c were all associated with higher CSM (P <0.05). The same results held true for ACM. The limitation of this article is that it is not possible to assess the effect of differences in systemic therapy on CSM in mPCa patients and clinical trials are needed to verify the results.

**Conclusions:**

For patients with mPCa, both RP and RT are beneficial to patients, and the efficacy of RP is better than RT from the perspective of CSM and ACM. Older age, higher gleason scores and the more advanced AJCC TNM stage all put patients at higher risk of dying.

**Patient summary:**

A large population-based cancer database showed that in addition to first-line therapy (hormonal treatment), RP and radiotherapy can also benefit patients with mPCa.

## 1 Introduction

Prostate cancer is the second most commonly diagnosed cancer and the sixth leading cause of cancer death among men worldwide (1). In 2022, the number of estimated new cases of prostate cancer in the United State is 268490, and the number of estimated deaths is 34500 (2). Although prostate cancer is an indolent tumor, many patients progress to intermediate or high-risk localized, locally advanced, or metastatic cancer.

RP and RT are important options in the principle treatments of localized prostate cancer (3). For locally advanced disease and metastatic prostate cancer, continuous androgen deprivation therapy (ADT) is the first line of treatment (4–6). However, whether ADT in combination with local treatment (RP or radiotherapy) for primary tumor will benefit patients remains controversial.

Currently, RT is usually reserved for symptomatic lesions for mPCa. However, several studies have shown systemic benefits of RT in addition to local symptom control, such as reducing tumor oxygenation leading to tumor cell apoptosis and necrosis (7). RP has been shown to be feasible and safe for men with mPCa, although the survival benefit is less certain (8).

In two previous retrospective studies based on SEER database, both RP and RT improved CSM in patients with mPCa, but one of them lacked propensity score matching, and the other had a limited sample size of treatment group (RP: 313 patients, RT:161 patients) and ignored the effect of local treatment on non-cancer-specific mortality or all-cause mortality (9, 10). Another retrospective analytic cohort study also showed that prostate RT was associated with improved OS (11). These studies were limited to retrospective analysis and were plagued by sample size. Interestingly, two randomized-controlled-trials (RCTs) (STAMPEDE and HORRAD) showed that RT in patients with metastatic prostate cancer did not improve OS (7, 12). In addition, multiple studies have suggested an OS benefit and lower CSM for RP in mPCa (13, 14).

In this study, we enrolled latest patients with mPCa and compared the association of different local treatments with CSM, and OS in competing risk regression analysis and multivariable cox regression analysis after propensity score matching. Additionally, we have innovatively identified the effect of EBRT on CSM and OS in metastatic prostate cancer.

## 2 Methods

### 2.1 Patient selection

20098 patients with M1a-M1c (sixth edition of American Joint Committee on Cancer [AJCC] Cancer Staging Manual) metastatic prostate cancer who excluded autopsy and death certificate from reported sources were identified from the SEER (18 registers, 2000-2018) database (15). Complete follow-up data and positive histology were ensured for each patient. Of these patients, 19433 did not receive local therapy and 665 received local treatment, including 377 who underwent RP (surgery site code 50, 70 and 80) and 288 who received RT (including brachytherapy, radioisotopes and combination of beam with implants or isotopes). Age, race, Gleason score, prostate-specific antigen (PSA) values and AJCC TNM staging of each patient were included in the analysis.

Patients were stratified based on RP or RT or NLT. Patients treated with beam radiation were excluded based on the lack of beam radiation organ site-specific records within SEER. Patients treated with endoscopic therapy were also excluded. Incidentally, when patients receiving beam radiation were included in the analysis, we found that radiotherapy (for *in situ* or metastases) (HR 1.06, 95%CI 1.00-1.11) slightly increased the CSM of patients with mPCa.

### 2.2 Propensity score matching

The sample matching between the NLT and LT was achieved *via* the “MatchIt” packages in R software (ratio 1:1), which based on the nearest-neighbor matching (16). Characteristics used to calculate propensity scores included age, race, year of diagnosis, Gleason score, PSA value, AJCC.T, AJCC.N, and AJCC.M stage, which have been found to be independent risk factors in previous studies (17). Following matching, the treatment effect was evaluated in subsequent analyses between NLT (n= 665) and LT (n= 665) who were successfully matched. Similarly, PSM (ratio 1:1) also identified suitable samples of RP (n= 288) and RT (n= 288) for analysis.

### 2.3 Establishment of nomogram

The multivariable Cox regression analysis was performed to screen the indicators affecting OS and predict their weights. By using the “rms” R packages, a nomogram with the independent indicators such as age, race, Gleason score, PSA value, AJCC.T, AJCC.N, AJCC.M stage, RP and RT was established for predicting OS in mPCa (18).

### 2.4 Survival analysis

The differences in OS between the NLT, RP and RT were represented by the Kaplan-Meier curve using the “survival” R package (19).

### 2.5 Statistical analysis

Competing risk regression analysis was used to calculate the cumulative incidence of CSM and independent factors associated with CSM were identified by using stepwise multivariable competing risk regression analysis (20). Pearson chi-square analysis was used to determine variables differing among NLT and LT. Statistical significance was defined as p < 0.05. All statistical analyses were conducted using R 4.1.2 (https://www.r-project.org/).

## 3 Results

### 3.1 mPCa benefits from LT

The median age of 19433 patients without local treatment was 71 years, compared with RP (63 years) and RT (67 years) (**Table 1**). According to Gleason scores, the highest proportion of NLT was Gleason score = 7 (34.6%), corresponding to LT of 29.3%, but the rate for Gleason score ≥ 8 of NLT (6%) was lower than LT (14.3%). The proportion of PSA values > 20 (40.6%) was significantly higher than those with a PSA value ≤ 20 (10.4%) in patients with NLT, whereas the opposite composition was observed in patients with LT (PSA > 20: 18.0%, PSA ≤ 20:30.2%). The rate for AJCC.T stage T3-T4 (55.5%) was higher than T1-T2 (21.6%) in NLT patients, and the same was true for LT patients (T1-T2: 9.8%, T3-T4: 50.8%). As can be seen in the AJCC.N staging, the ratio of N0 was much higher than that of N1 in both NLT (N0: 51.9%, N1: 24.0%) and LT (N0: 62.6%, N1: 25.1%) patients. Finally, in AJCC.M staging, NLT patients were composed as follows, M1a: 6%, M1b: 68.6%, M1c: 21.3%, while LT patients were composed as follows, M1a: 11.1%, M1b: 65.6%, M1c: 20.0%. Specific to the classification under LT patients, there was a roughly consistent trend in the composition of Gleason score, AJCC.T stage, AJCC.N stage and AJCC.M stage between RP and RT. The difference was PSA value, with RP patients having the higher proportion of PSA ≤ 20 (41.1%) and RT patients having the higher proportion of PSA > 20 (23.3%). In a total of 20098 enrolled patients with mPCa, the number of cancer-specific deaths in NLT, RP and RT were 12489, 111, 146, respectively.

**Table 1:** Characteristics of patients with and without propensity score matching.

Variables	No local treatment (n = 19433;%)	Local treatment (n = 665;%)	P value ^a^	Propensity score-adjusted no local treatment (n = 665;%)	Propensity score-adjusted local treatment (n = 665;%)	P value ^b^
Median age, yr (IQR)	71.00 (62.00, 79.00)	64.00 (58.00, 70.00)	<0.001	63.00 (57.00, 70.00)	64.00 (58.00, 70.00)	0.346
Race, n (%)			0.349			0.923
White	13943 (71.7)	497 (74.7)		486 (73.1)	497 (74.7)	
African American	4200 (21.6)	128 (19.2)		137 (20.6)	128 (19.2)	
Other	1225 (6.3)	37 (5.6)		39 (5.9)	37 (5.6)	
Unknown	65 (0.3)	3 (0.5)		3 (0.5)	3 (0.5)	
Year of diagnosis, n (%)			0.601			0.784
2004	1265 (6.5)	41 (6.2)		51 (7.7)	41 (6.2)	
2005	1308 (6.7)	46 (6.9)		56 (8.4)	46 (6.9)	
2006	1354 (7.0)	56 (8.4)		58 (8.7)	56 (8.4)	
2007	1394 (7.2)	46 (6.9)		49 (7.4)	46 (6.9)	
2008	1454 (7.5)	55 (8.3)		55 (8.3)	55 (8.3)	
2009	1485 (7.6)	51 (7.7)		52 (7.8)	51 (7.7)	
2010	1606 (8.3)	53 (8.0)		61 (9.2)	53 (8.0)	
2011	1619 (8.3)	63 (9.5)		57 (8.6)	63 (9.5)	
2012	1734 (8.9)	48 (7.2)		31 (4.7)	48 (7.2)	
2013	1905 (9.8)	55 (8.3)		49 (7.4)	55 (8.3)	
2014	2022 (10.4)	79 (11.9)		75 (11.3)	79 (11.9)	
2015	2287 (11.8)	72 (10.8)		71 (10.7)	72 (10.8)	
Gleason score, n (%)			<0.001			0.291
≤6	196 (1.0)	28 (4.2)		33 (5.0)	28 (4.2)	
7	6732 (34.6)	195 (29.3)		178 (26.8)	195 (29.3)	
≥8	1171 (6.0)	95 (14.3)		79 (11.9)	95 (14.3)	
Unknown	11334 (58.3)	347 (52.2)		375 (56.4)	347 (52.2)	
PSA, ng/ml, n (%)			<0.001			0.68
>20	7895 (40.6)	120 (18.0)		118 (17.7)	120 (18.0)	
≤20	2013 (10.4)	201 (30.2)		188 (28.3)	201 (30.2)	
Unknown	9525 (49.0)	344 (51.7)		359 (54.0)	344 (51.7)	
AJCC T stage, n (%)			<0.001			0.71
T1-T2	4199 (21.6)	60 (9.0)		65 (9.8)	60 (9.0)	
T3-T4	10792 (55.5)	329 (49.5)		338 (50.8)	329 (49.5)	
TX	4442 (22.9)	276 (41.5)		262 (39.4)	276 (41.5)	
AJCC N stage, n (%)			<0.001			0.372
N0	10086 (51.9)	416 (62.6)		440 (66.2)	416 (62.6)	
N1	4672 (24.0)	167 (25.1)		148 (22.3)	167 (25.1)	
NX	4675 (24.1)	82 (12.3)		77 (11.6)	82 (12.3)	
AJCC M stage, n (%)			<0.001			0.991
M1a	1165 (6.0)	73 (11.0)		74 (11.1)	73 (11.0)	
M1b	13379 (68.8)	436 (65.6)		438 (65.9)	436 (65.6)	
M1c	4143 (21.3)	133 (20.0)		132 (19.8)	133 (20.0)	
M1NOS	746 (3.8)	23 (3.5)		21 (3.2)	23 (3.5)	
Cancer-specific death, n (%)	12489 (NA)	257 (NA)	NA	397 (NA)	257 (NA)	NA

IQR, interquartile range; AJCC, American Joint Committee on Cancer; NLT, no local treatment; LT, local treatment; NA, not applicable;

PSA, prostate specific antigen.

^a^Comparing NLT versus LT (unmatched).

^b^Comparing NLT versus LT (propensity score-adjusted cohorts).

proportions presented are of the corresponding subgroups.

Following propensity score matching, there was no statistical differences in all clinical characteristics between the NLT and LT patients, but residual statistically significantly differences remained for age, PSA value, AJCC.T stage and AJCC.N stage between RP and RT patients. After propensity score matching, the number of cancer-specific deaths in the NLT, RP, RT groups were 397, 87 and 146, respectively, and the CSM rate of NLT, RP, RT were 59.7%, 30.2% and 50.7%, respectively (**Table 1**).

**Figure 1:**
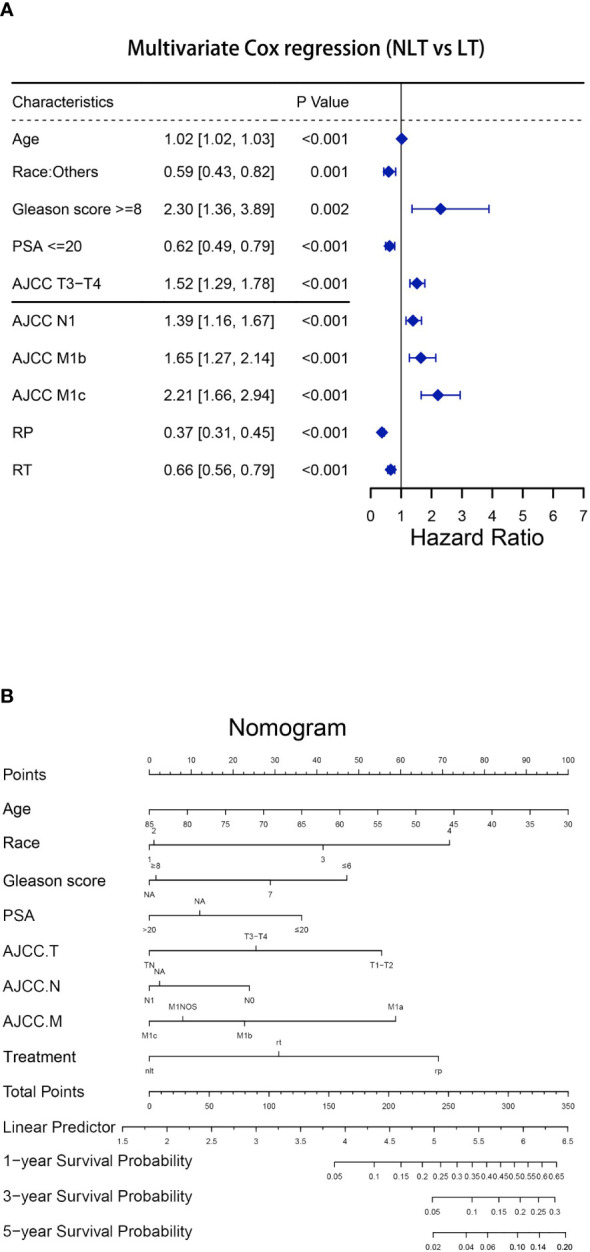
Establishment of the nomogram to predict the survival for patients with mPCa (NLT vs LT). **(A)**Multivariate Cox regression analysis of patients with mPCa for independent risk factors (NLT vs LT). **(B)** Establishment of the nomogram predicting survival of patients with mPCa (NLT vs LT).

In a multivariable competing risk regression analysis after PSM, both RP (HR 0.36, 95%CI 0.29-0.45, P<0.001) and RT (HR 0.77, 95%CI 0.63-0.95, P<0.05) had lower CSM rate compared to NLT (**Table 2**). In addition to no local treatments that could increase CSM, the white race (vs Asian or Pacific Islander: HR 0.689, 95%CI 0.49-0.97), Gleason score ≥ 8 (vs Gleason ≤ 6, HR 2.06, 95%CI 1.12-3.80, P< 0.05), PSA value >20 (vs PSA ≤ 20, HR 1.38 95%CI 1.06-1.80, P< 0.05), T3-T4 (vs T1-T2, HR 1.64, 95%CI 1.37-1.97, P< 0.001), N1 (vs N0, HR 1.43, 95%CI 1.16-1.76, P< 0.001) and M1b (vs M1a, HR 1.90, 95%CI 1.39-2.58, P< 0.001) - M1c (vs M1a, HR 2.46, 95%CI 1.76-3.43, P< 0.001) were associated with higher CSM. Besides CSM, OS was also a clinically non-negligible prognostic indicator. A multivariable Cox regression analysis after PSM drew conclusions close to competing risk regression analysis, older age (HR 1.02, 95%CI 1.02-1.03, P< 0.001), Gleason ≥ 8 (HR 2.30, 95%CI 1.36-3.89, P< 0.05), T3-T4 (HR 1.52, 95%CI 1.29-1.78, P<0.001), N1 (HR 1.39, 95%CI 1.16-1.67, P< 0.001), M1b (HR 1.65, 95%CI 1.27-2.14, P< 0.001) and M1c (HR 2.21, 95%CI 1.66-2.94, P<0.001) impaired OS, while Asian or Pacific Islander (HR 0.59,95%CI 0.43-0.82, P<0.05), PSA ≤ 20 (HR 0.62, 95%CI 0.49-0.79, P<0.001), RP (HR 0.37, 95%CI 0.31-0.45, P<0.001) and RT (HR 0.66, 95%CI 0.56-0.79, P<0.001) improved OS of patients with mPCa (**Figure 1A**). We then constructed a nomogram to predict 1-year, 3-year, and 5-year OS using independent prognostic indictors (**Figure 1B**).

**Table 2:** Multivariable competing risks regression analysis after PSM of patients with metastatic prostate cancer, stratified according to treatment type (NLT vs LT).

Variables	Local treatment versus no local treatment	
	HR (95% CI)	p value
Type of treatment		
**No local therapy**	**Ref.**	
**Radiotherapy**	**0.77 (0.63-0.95)**	**0.01**
**Radical prostatectomy**	**0.36 (0.29-0.45)**	**<0.001**
**Age (yr)**	1.00 (1.00-1.02)	0.12
Race		
**White**	**Ref.**	
**African American**	0.88 (0.71-1.07)	0.20
**Other**	**0.69 (0.49-0.97)**	**0.03**
Year of diagnosis		
**2004**	**Ref.**	
**2005**	0.87 (0.59-1.28)	0.47
**2006**	0.93 (0.62-1.38)	0.72
**2007**	1.10 (0.73-1.66)	0.65
**2008**	1.14 (0.78-1.67)	0.51
**2009**	1.28 (0.87-1.86)	0.21
**2010**	0.83 (0.47-1.45)	0.51
**2011**	1.00 (0.58-1.73)	0.99
**2012**	1.31 (0.69-2.47)	0.41
**2013**	0.75 (0.41-1.39)	0.36
**2014**	0.61 (0.34-1.11)	0.11
**2015**	0.69 (0.38-1.25)	0.22
Gleason score		
**≤6**	**Ref.**	
**7**	1.42 (0.76-2.68)	0.27
**≥8**	**2.06 (1.12-3.80)**	**0.02**
PSA		
**≤20**	**Ref.**	
**>20**	**1.38 (1.06-1.80)**	**0.01**
AJCC.T		
**T1-T2**	**Ref.**	
**T3-T4**	**1.64 (1.37-1.97)**	**<0.001**
AJCC.N		
**N0**	**Ref.**	
**N1**	**1.43 (1.16-1.76)**	**<0.001**
AJCC.M		
**M1a**	**Ref.**	
**M1b**	**1.90 (1.39-2.58)**	**<0.001**
**M1c**	**2.46 (1.76-3.43)**	**<0.001**
**M1NOS**	**2.85 (1.66-4.90)**	**<0.001**

CI, confidence interval; HR, hazard ratio; Ref., reference;

AJCC, American Joint Committee on Cancer. The meaning of the bold values is p<0.05.

Interestingly, in the initial analysis, we found that CSM of radiotherapy (including beam radiation: for *in situ* or metastases) (HR 1.06, 95%CI 1.00-1.11, P< 0.05) was slightly elevated compared to non-treatment, possibly suggesting that beam radiation for metastatic prostate cancer, especially for metastases, may not be beneficial in improving CSM but rather increase the risk of cancer-specific death (**Tables S1**, **S2**). However, no statistically significant difference was found between radiotherapy (including beam radiation: for *in situ* or metastases) (HR 0.97, 95%CI 0.93-1.02, P= 0.24) and non-treatment in multivariate Cox regression analysis after PSM, while RP (HR 0.38, 95%CI 0.33-0.45, P<0.001) still substantially improved OS (**Figure S1**).

### 3.2 RP is superior to RT in mPCa

**Figure 2:**
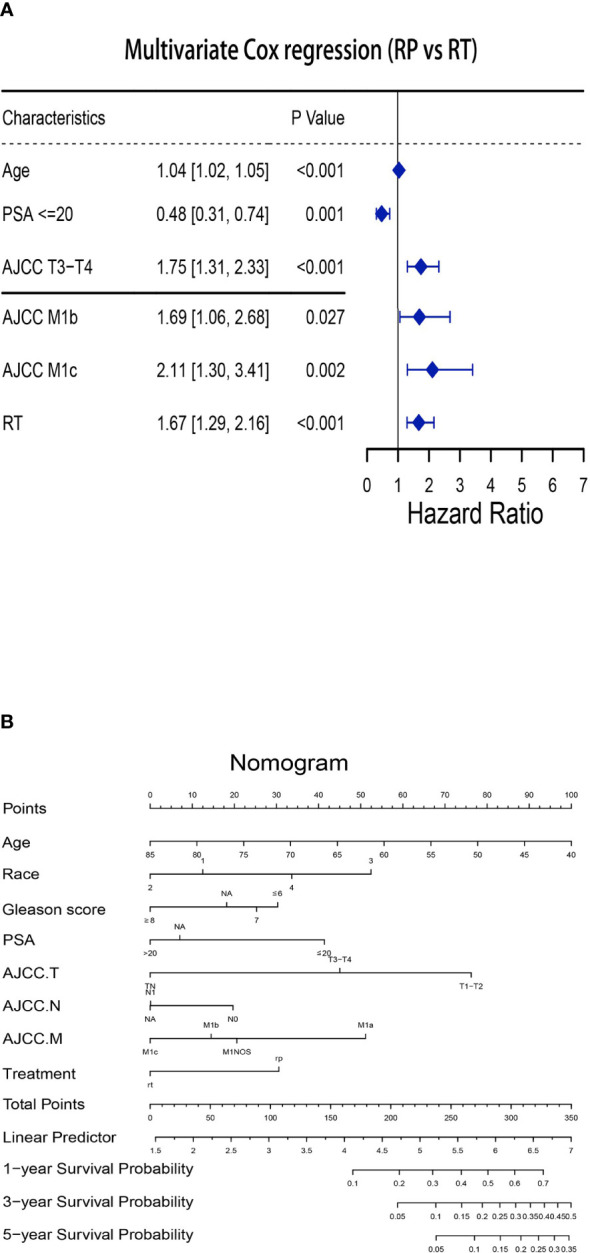
Establishment of the nomogram to predict the survival for patients with mPCa (RP vs RT). **(A)** Multivariate Cox regression analysis of patients with mPCa for independent risk factors (RP vs RT). **(B)** Establishment of the nomogram predicting survival of patients with mPCa (RP vs RT).

After confirming that local treatment was helpful in improving CSM and OS in patients, we further investigated the differences between RP and RT. Obviously, a competing risk regression (RP vs RT) after PSM showed that RP (HR 0.56, 95%CI 0.41-0.76, P< 0.001) highlighted therapeutic advantages over RT (**Tables 3**, **4**). There was no difference between races in improving CSM when RP versus RT. Additionally, CSM was also higher in presence of PSA> 20 (vs PSA≤ 20: HR 2.07, 95%CI 1.27-3.38, P< 0.05), T3-T4 (vs T1-T2: HR 1.78, 95%CI 1.25-2.54, P< 0.05), N1 (vs N0: HR 1.58, 95%CI 1.11-2.25, P< 0.05) and M1b (vs M1a: HR 2.22, 95%CI 1.25-3.93, P< 0.05)-M1c (vs M1a: HR 3.11, 95%CI 1.72-5.63, P<0.001). A multivariate Cox regression after PSM (RP vs RT) was performed to determine independent factors affecting patients OS (**Figure 2A**). The results showed that prognosis was worse in patients with older age (HR 1.04, 95%CI 1.02-1.05, P< 0.001), T3-T4 (HR 1.75, 95%CI 1.31-2.33, P< 0.001) and M1b (HR 1.69, 95%CI 1.06-2.68, P< 0.05)-M1c (HR 2.11, 95%CI 1.30-3.41, P<0.05). Similarly, a nomogram was plotted to predict 1-year, 3-year, and 5-year OS with mPCa who underwent RP or RT (**Figure 2B**).

**Table 3:** Characteristics of LT patients with and without propensity score matching.

Variables	Radical prostatectomy (n = 377;%)	Radiotherapy (n = 288;%)	P value ^a^	Propensity score-adjusted radical prostatectomy (n = 288;%)	Propensity score-adjusted radiotherapy (n = 288;%)	P value ^b^
Median age, yr (IQR)	63.00 (57.00, 68.00)	67.00 (60.00, 74.00)	<0.001	63.00 (58.00, 68.00)	67.00 (60.00, 74.00)	<0.001
Race, n (%)			0.071			0.52
White	296 (78.5)	201 (69.8)		215 (74.7)	201 (69.8)	
African American	61 (16.2)	67 (23.3)		56 (19.4)	67 (23.3)	
Other	18 (4.8)	19 (6.6)		15 (5.2)	19 (6.6)	
Unknown	2 (0.5)	1 (0.3)		2 (0.7)	1 (0.3)	
Year of diagnosis, n (%)			<0.001			0.428
2004	14 (3.7)	27 (9.4)		14 (4.9)	27 (9.4)	
2005	25 (6.6)	21 (7.3)		24 (8.3)	21 (7.3)	
2006	23 (6.1)	33 (11.5)		22 (7.6)	33 (11.5)	
2007	23 (6.1)	23 (8.0)		21 (7.3)	23 (8.0)	
2008	28 (7.4)	27 (9.4)		26 (9.0)	27 (9.4)	
2009	28 (7.4)	23 (8.0)		25 (8.7)	23 (8.0)	
2010	29 (7.7)	24 (8.3)		22 (7.6)	24 (8.3)	
2011	34 (9.0)	29 (10.1)		28 (9.7)	29 (10.1)	
2012	35 (9.3)	13 (4.5)		18 (6.2)	13 (4.5)	
2013	39 (10.3)	16 (5.6)		20 (6.9)	16 (5.6)	
2014	46 (12.2)	33 (11.5)		37 (12.8)	33 (11.5)	
2015	53 (14.1)	19 (6.6)		31 (10.8)	19 (6.6)	
Gleason score, n (%)			<0.001			0.156
≤6	17 (4.5)	11 (3.8)		15 (5.2)	11 (3.8)	
7	122 (32.4)	73 (25.3)		75 (26.0)	73 (25.3)	
≥8	68 (18.0)	27 (9.4)		42 (14.6)	27 (9.4)	
Unknown	170 (45.1)	177 (61.5)		156 (54.2)	177 (61.5)	
PSA, ng/ml, n (%)			<0.001			0.001
>20	53 (14.1)	67 (23.3)		51 (17.7)	67 (23.3)	
≤20	155 (41.1)	46 (16.0)		84 (29.2)	46 (16.0)	
Unknown	169 (44.8)	175 (60.8)		153 (53.1)	175 (60.8)	
AJCC T stage, n (%)			<0.001			<0.001
T1-T2	16 (4.2)	44 (15.3)		16 (5.6)	44 (15.3)	
T3-T4	138 (36.6)	191 (66.3)		138 (47.9)	191 (66.3)	
TX	223 (59.2)	53 (18.4)		134 (46.5)	53 (18.4)	
AJCC N stage, n (%)			<0.001			<0.001
N0	224 (59.4)	192 (66.7)		184 (63.9)	192 (66.7)	
N1	127 (33.7)	40 (13.9)		79 (27.4)	40 (13.9)	
NX	26 (6.9)	56 (19.4)		25 (8.7)	56 (19.4)	
AJCC M stage, n (%)			0.019			0.101
M1a	50 (13.3)	23 (8.0)		40 (13.9)	23 (8.0)	
M1b	252 (66.8)	184 (63.9)		180 (62.5)	184 (63.9)	
M1c	66 (17.5)	67 (23.3)		59 (20.5)	67 (23.3)	
M1NOS	9 (2.4)	14 (4.9)		9 (3.1)	14 (4.9)	
Cancer-specific death, n (%)	111 (NA)	146 (NA)	NA	87 (28.2)	146 (66.5)	NA

IQR, interquartile range; AJCC, American Joint Committee on Cancer; NLT, no local treatment; LT, local treatment; NA, not applicable; PSA, prostate specific antigen.

^a^Comparing RP versus RT (unmatched).

^b^Comparing RP versus RT (propensity score-adjusted cohorts).

proportions presented are of the corresponding subgroups.

**Table 4:** Multivariable competing risks regression analysis after PSM of patients with metastatic prostate cancer, stratified according to treatment type (RP vs RT).

Variables	Radical prostatectomy versus radiotherapy	
	HR (95% CI)	p value
Type of treatment		
**Radiotherapy**	**Ref.**	
**Radical prostatectomy**	**0.56 (0.41-0.76)**	**<0.001**
**Age (yr)**	**1.03 (1.02-1.05)**	**<0.001**
Race		
**White**	**Ref.**	
**African American**	1.40 (0.99-1.87)	0.058
**Other**	0.59 (0.35-1.01)	0.054
Year of diagnosis		
**2004**	**Ref.**	
**2005**	0.84 (0.44-1.63)	0.61
**2006**	0.64 (0.31-1.30)	0.22
**2007**	1.01 (0.50-2.01)	0.98
**2008**	1.07 (0.57-2.01)	0.84
**2009**	1.16 (0.60-2.23)	0.67
**2010**	0.93 (0.36-2.39)	0.88
**2011**	1.63 (0.69-3.86)	0.27
**2012**	1.67 (0.65-4.34)	0.29
**2013**	0.92 (0.36-2.36)	0.86
**2014**	0.85 (0.35-2.09)	0.73
**2015**	1.15 (0.46-2.92)	0.76
Gleason score		
**≤6**	**Ref.**	
**7**	1.08 (0.32-3.65)	0.91
**≥8**	2.29 (0.75-6.96)	0.15
PSA		
**≤20**	**Ref.**	
**>20**	**2.07 (1.27-3.38)**	**0.0035**
AJCC.T		
**T1-T2**	**Ref.**	
**T3-T4**	**1.78 (1.25-2.54)**	**0.0014**
AJCC.N		
**N0**	**Ref.**	
**N1**	**1.58 (1.11-2.25)**	**0.011**
AJCC.M		
**M1a**	**Ref.**	
**M1b**	**2.22 (1.25-3.93)**	**0.006**
**M1c**	**3.11 (1.72-5.63)**	**<0.001**
**M1NOS**	**2.84 (1.26-6.42)**	**0.012**

CI, confidence interval; HR, hazard ratio; Ref., reference; AJCC, American Joint Committee on Cancer. The meaning of the bold values is p<0.05.

**Figure 3:**
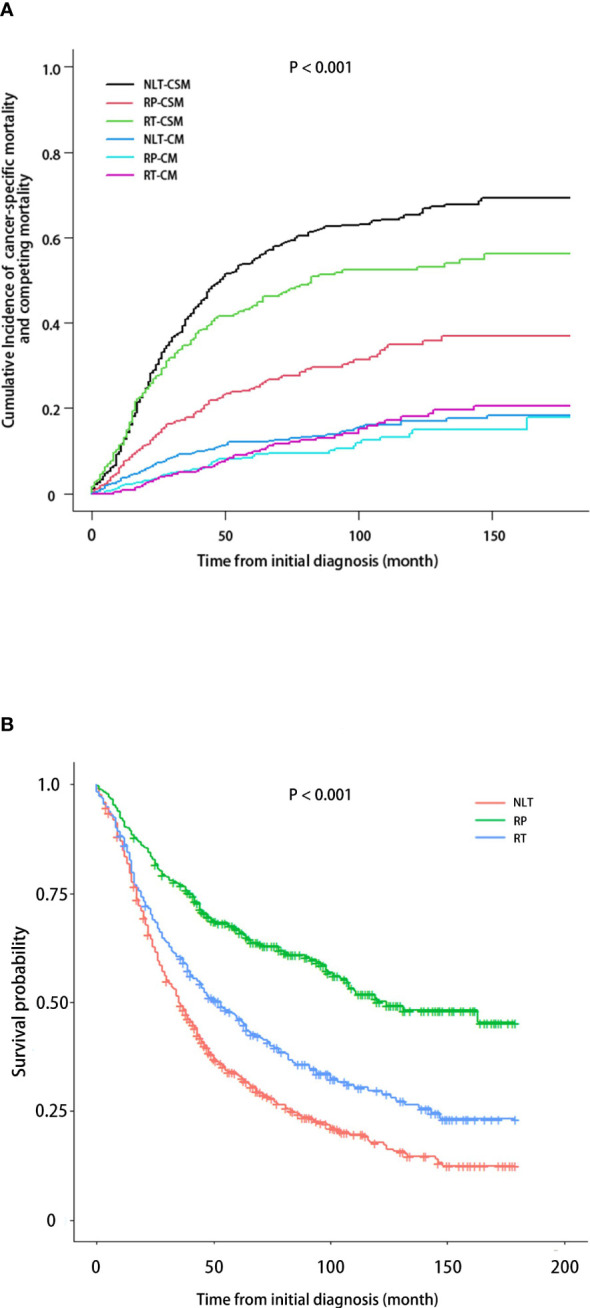
CSM and OS in patients with mPCa based on treatment received. **(A)** Cumulative incidence of CSM, accounting for the competing risk of non-PCa. **(B)** Kaplan-Meier analysis demonstrating OS.

The median follow-up of 1330 patients enrolled after PSM was 42 months (IQR, 20.00-76.75). A cumulative incidence curve of CSM and competing mortality was presented in **Figure 3A**. Compared with NLT, RP could reduce both of CSM and competing mortality of patients, and the efficacy of reducing CSM was particularly significant, while RT could slightly reduce CSM of patients, but had no advantage in improving mortality from other causes. **Figure 3B** revealed the differences in OS between NLT, RP and RT. Consistent with above conclusion, both RP and RT could improve the OS of patients with mPCa, with RP being better.

## 4 Discussion

For metastatic prostate cancer, continuous androgen deprivation therapy remains the most effective treatment. In recent years, increasing studies have focused on ADT combined with other therapies to further improve patient outcomes (5, 21). Several large RCTs have been conducted with ADT combined with chemotherapy agents and combined with the new hormonal treatments (such like abiraterone and enzalutamide), and the data showed that the combination therapy was truly effective in improving OS (4, 22, 23). However, the significance of local treatment for metastatic prostate cancer remains unclear. Of note, in a randomized controlled phase 3 trial (STAMPEDE), radiotherapy improved failure-free survival in the standard of care group compared with standard of care plus radiotherapy (HR 0.76, 95% CI 0.68–0.84, P< 0·0001), but did not improve OS (HR 0.92, 95%CI 0.80–1.06; P= 0·266) (12). A similar conclusion to this study is the HORRAD trial, a multicenter RCT recruiting 432 patients with prostate-specific antigen (PSA) >20 ng/ml and primary bone mPCa (7). This trail showed no significant difference in OS (HR 0.90, 95% CI: 0.70–1.14, P = 0.4). These studies only addressed the effect of local treatment of RP on OS and ignored differences in CSM. However, a retrospective study of 13,692 patients with metastatic prostate cancer from 2004 to 2013 based on the SEER database showed that RP significantly reduced CSM (HR 0.48, 95% CI 0.35-0.66) compared with NLT (9). Similarly, another retrospective study of 8185 prostate cancer patients from 2004 to 2010 based on the SEER database showed that RP reduced CSM (HR 0.68, 95%CI 0.49-0.93), but the researcher did not perform propensity score matching on the patients prior to analysis (10). We could see that the above studies came to seemingly opposite conclusions, and it was clear that these studies only focused on the CSM or OS of patients without analyzing them individually, and both retrospective studies were limited to small sample size. Through these studies we remained in doubt as to whether patients with mPCa should receive local radiation therapy. A trial of 1538 patients with metastatic prostate cancer between 1998 and 2010 based on Munich Cancer Registry (MCR) showed that RP was effective in improving OS, but this study lacked of analysis of CSM and only 5% of the patients in this study received RP (14). In addition, a retrospective cohort study included 1809 men with biopsy Gleason score 9-10 prostate cancer indicated that no significant differences in CSM and OS were found between men treated with ERBT or RP (24). In summary, these studies are insufficient for us to draw robust conclusion. Our work is the first to compare the effects of different local treatment on CSM and OS in patients with mPCa, while considering the impact of EBRT on patient outcomes.

Our study was conducted on the basis of eliminating the clinical characteristics that differed between NLT and LT, attributing the difference to then variable of treatment. Moreover, we were not limited to analyzing OS of patients, we also performed an analysis of CSM. After including the latest patients with metastatic prostate cancer, we noted that, consistent with previous retrospective studies, both RP and RT reduced CSM. Besides, both RP and RT improved OS. We found that clinical characteristics independently associated with increased CSM in patients who received local therapy included older age, higher PSA value, higher Gleason score, and more advanced AJCC.TNM staging (25, 26). Obviously, the existing research evidence suggested that these factors are directly related to the worse prognosis of patients, and in line with the fact that the patients with these features also benefit less from RP and RT, suggesting that we should aggressively perform local treatment earlier for low-risk prostate cancer patients to reap greater benefits. Also, with full consideration for the patients’ basal physical condition, active administration of RP will obtain greater benefits compared with RT. However, it cannot be ignored that in specific cases, the choice of treatment should also consider the feasibility of surgery, the sequelae of surgery, the quality of life of patients and so on (27).

Interestingly, we got some noteworthy findings in the initial analysis. Despite the belief that EBRT could alleviate symptoms, patients receiving EBRT even had a slightly elevated CSM compared with NLT, and there was no significant difference in OS. In contrast to Amar U. Kishan et al., RP still benefited patients in the long term despite Gleason score ≥ 8 (4). This result raised a new question about whether EBRT was a wise choice for patients with mPCa. Clearly, for patients with mPCa, ADT combined with RP was superior to ADT combined with local brachytherapy, and EBRT was the least favorable option.

Our research also has many unavoidable limitations. First, our study is only a retrospective study. Second, the data we used were all from SEER database, variables unavailable from SEER undoubtedly limited our analysis. Third, it can be seen that many missing values appeared in the process of our analysis, especially the PSA value, which in turn is a credible independent risk factor (28). Fourth, the SEER database does not provide information on the specific extent of metastasis, which affects the prognosis and efficacy of the patient. Finally, the SEER database lacks EBRT coding for specific sites, which may lead to the inclusion of some patients in the wrong treatment stratification, thereby reducing the reliability of the findings.

## 5 Conclusion

Our study demonstrated that LT can improve CSM and OS in patents with mPCa compared with NLT, and RP has more advantages in reducing CSM and improving OS than RT. Younger age, lower PSA value, lower Gleason score and clinical stage are associated with a greater benefit for LT. And similarly, these patients are associated with a greater benefit for RP than for RT. In addition, we provide evidence that EBRT even slightly elevated CSM compared with NLT, while OS was not statistically different.

## Data availability statement

The original contributions presented in the study are included in the article/**Supplementary Material**. Further inquiries can be directed to the corresponding author.

## Author contributions

Conceptualization, QM, CL and JC. Data curation, ZW. Formal analysis, ZW, QM. Funding acquisition, XZ and HR. Investigation, CL and CY. Methodology, CL and YY. Project administration, QM, ZW and ZS. Resources, YY and KC. Software, QM, ZW. Supervision, YZ. Validation, HR and XZ. Visualization, GC. Writing – original draft, QM. Writing – review & editing, XZ. All authors contributed to the article and approved the submitted version.

## Supplementary material

Supplementary Figure 1 Establishment of the nomogram to predict the survival for patients with mPCa (NLT vs RP/BR). **(A)** Multivariate Cox regression analysis of patients with mPCa for independent risk factors (NLT vs RP/BR). **(B)** Establishment of the nomogram predicting survival of patients with mPCa (NLT vs RP/BR).
