# Supplementary figures and images for: Overall survival and cancer-specific survival were improved in local treatment of metastatic prostate cancer
Source: Front Oncol. 2023 May 3;13:1130680. doi: 10.3389/fonc.2023.1130680 (PMC10189015; doi:10.3389/fonc.2023.1130680)

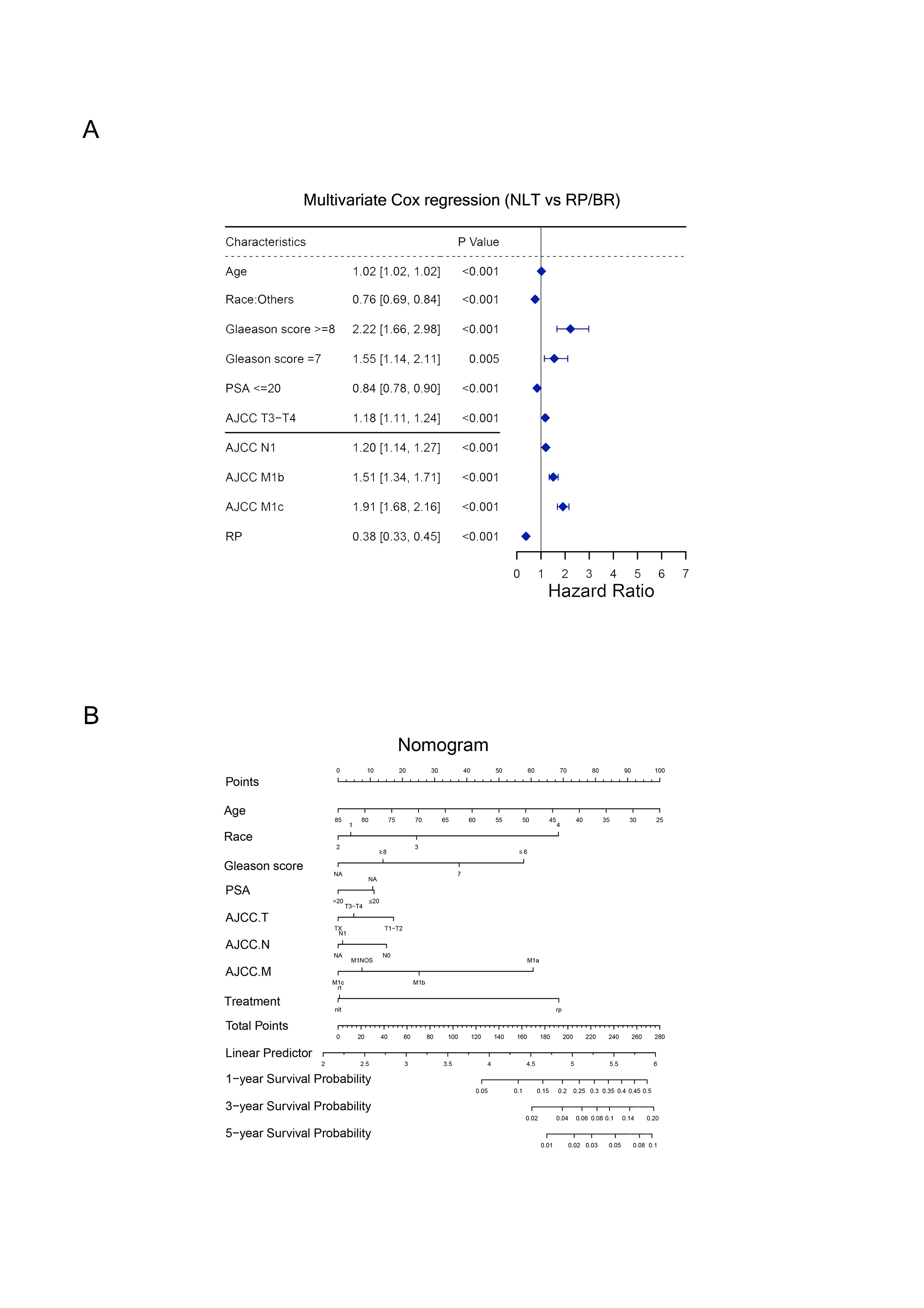

Supplement: Supplementary Figure 1 — Establishment of the nomogram to predict the survival for patients with mPCa (NLT vs RP/BR). (A) Multivariate Cox regression analysis of patients with mPCa for independent risk factors (NLT vs RP/BR). (B) Establishment of the nomogram predicting survival of patients with mPCa (NLT vs RP/BR). [file Image_1.jpeg]
