# Supplementary material for: Overall survival and cancer-specific survival were improved in local treatment of metastatic prostate cancer
Source: Front Oncol. 2023 May 3;13:1130680. doi: 10.3389/fonc.2023.1130680 (PMC10189015; doi:10.3389/fonc.2023.1130680)
Supplement: Supplementary file 2 [file Table_1.docx]

Table S1 – Comparation of NLT versus RP and/or BR with and without propensity score matching

| Variables | No radical prostatectomy and beam radiation  (n = 14979；%) | Radical prostatectomy and/or beam radiation（n = 5119；%） | P value ^a^ | Propensity  score-adjusted  no radical prostatectomy and beam radiation  （n = 5119；%） | Propensity score-adjusted radical prostatectomy and/or beam radiation  (n = 5119；%) | P value ^b^ |
| --- | --- | --- | --- | --- | --- | --- |
| Median age, yr (IQR) | **72.0 (63.0, 80.0)** | **67.0 (60.0, 76.0)** | **<0.001** | **67.0 (60.0, 76.0)** | **67.0 (60.0, 76.0)** | **0.865** |
| Race, n (%) |  |  | **0.234** |  |  | **0.206** |
| White | **10753 (71.8)** | **3687 (72.0)** |  | **3767 (73.6)** | **3687 (72.0)** |  |
| African American | **3231 (21.6)** | **1097 (21.4)** |  | **1042 (20.4)** | **1097 (21.4)** |  |
| Other | **937 (6.3)** | **325 (6.3)** |  | **305 (6.0)** | **325 (6.3)** |  |
| Unknown | **58 (0.4)** | **10 (0.2)** |  | **5 (0.1)** | **10 (0.2)** |  |
| Year of diagnosis, n (%) |  |  | **0.276** |  |  | **0.995** |
| 2004 | **1001 (6.7)** | **305 (6.0)** |  | **286 (5.6)** | **305 (6.0)** |  |
| 2005 | **1011 (6.7)** | **343 (6.7)** |  | **368 (7.2)** | **343 (6.7)** |  |
| 2006 | **1041 (6.9)** | **369 (7.2)** |  | **374 (7.3)** | **369 (7.2)** |  |
| 2007 | **1087 (7.3)** | **353 (6.9)** |  | **364 (7.1)** | **353 (6.9)** |  |
| 2008 | **1147 (7.7)** | **362 (7.1)** |  | **372 (7.3)** | **362 (7.1)** |  |
| 2009 | **1158 (7.7)** | **378 (7.4)** |  | **377 (7.4)** | **378 (7.4)** |  |
| 2010 | **1213 (8.1)** | **446 (8.7)** |  | **447 (8.7)** | **446 (8.7)** |  |
| 2011 | **1218 (8.1)** | **464 (9.1)** |  | **468 (9.1)** | **464 (9.1)** |  |
| 2012 | **1330 (8.9)** | **452 (8.8)** |  | **433 (8.5)** | **452 (8.8)** |  |
| 2013 | **1455 (9.7)** | **505 (9.9)** |  | **504 (9.8)** | **505 (9.9)** |  |
| 2014 | **1577 (10.5)** | **524 (10.2)** |  | **527 (10.3)** | **524 (10.2)** |  |
| 2015 | **1741 (11.6)** | **618 (12.1)** |  | **599 (11.7)** | **618 (12.1)** |  |
| Gleason score, n (%) |  |  | **0.008** |  |  | **0.188** |
| ≤6 | **157 (1.0)** | **67 (1.3)** |  | **52 (1.0)** | **67 (1.3)** |  |
| 7 | **5230 (34.9)** | **1697 (33.2)** |  | **1739 (34.0)** | **1697 (33.2)** |  |
| ≥8 | **906 (6.0)** | **360 (7.0)** |  | **320 (6.3)** | **360 (7.0)** |  |
| Unknown | **8686 (58.0)** | **2995 (58.5)** |  | **3008 (58.8)** | **2995 (58.5)** |  |
| PSA, ng/ml, n (%) |  |  | **<0.001** |  |  | **0.328** |
| >20 | **5996 (40.0)** | **2019 (39.4)** |  | **2071 (40.5)** | **2019 (39.4)** |  |
| ≤20 | **1455 (9.7)** | **759 (14.8)** |  | **711 (13.9)** | **759 (14.8)** |  |
| Unknown | **7528 (50.3)** | **2341 (45.7)** |  | **2337 (45.7)** | **2341 (45.7)** |  |
| AJCC T stage, n (%) |  |  | **<0.001** |  |  | **0.261** |
| T1-T2 | **3208 (21.4)** | **1051 (20.5)** |  | **1056 (20.6)** | **1051 (20.5)** |  |
| T3-T4 | **8431 (56.3)** | **2690 (52.5)** |  | **2756 (53.8)** | **2690 (52.5)** |  |
| TX | **3340 (22.3)** | **1378 (26.9)** |  | **1307 (25.5)** | **1378 (26.9)** |  |
| AJCC N stage, n (%) |  |  | **<0.001** |  |  | **0.755** |
| N0 | **7747 (51.7)** | **2755 (53.8)** |  | **2776 (54.2)** | **2755 (53.8)** |  |
| N1 | **3501 (23.4)** | **1338 (26.1)** |  | **1305 (25.5)** | **1338 (26.1)** |  |
| NX | **3731 (24.9)** | **1026 (20.0)** |  | **1038 (20.3)** | **1026 (20.0)** |  |
| AJCC M stage, n (%) |  |  | **<0.001** |  |  | **0.113** |
| M1a | **987 (6.6)** | **251 (4.9)** |  | **220 (4.3)** | **251 (4.9)** |  |
| M1b | **10246 (68.4)** | **3569 (69.7)** |  | **3648 (71.3)** | **3569 (69.7)** |  |
| M1c | **3127 (20.9)** | **1149 (22.4)** |  | **1129 (22.1)** | **1149 (22.4)** |  |
| M1NOS | **619 (4.1)** | **150 (2.9)** |  | **122 (2.4)** | **150 (2.9)** |  |
| Cancer-specific death, n (%) | **9551 (NA)** | **3195 (NA)** | **NA** | **3289 (NA)** | **3195 (NA)** | **NA** |
| IQR = interquartile range; AJCC = American Joint Committee on Cancer; NLT = No radical prostatectomy and beam radiation; NA = not applicable;  PSA = prostate specific antigen; BR = beam radiation  a Comparing NT versus radical prostatectomy and/or beam radiation (unmatched).  b Comparing NT versus radical prostatectomy and/or beam radiation (propensity score-adjusted cohorts).  proportions presented are of the corresponding subgroups. | | | | | | |
