# Supplementary material for: Overall survival and cancer-specific survival were improved in local treatment of metastatic prostate cancer
Source: Front Oncol. 2023 May 3;13:1130680. doi: 10.3389/fonc.2023.1130680 (PMC10189015; doi:10.3389/fonc.2023.1130680)
Supplement: Supplementary file 3 [file Table_2.docx]

Table S2 – Multivariable competing risks regression analysis after PSM of patients with metastatic prostate cancer, stratified according to treatment type (NLT versus RP versus RT/BR)

| Variables | Radical prostatectomy and/or beam radiation versus no radical prostatectomy and beam radiation | |
| --- | --- | --- |
|  | **HR (95% CI)** | **p value** |
| Type of treatment |  |  |
| No local therapy | **Ref.** |  |
| Radiotherapy | **1.06 (1.00-1.11)** | **0.031** |
| Radical prostatectomy | **0.39 (0.32-0.47)** | **<0.001** |
| Age (yr） | **1.00 (1.00-1.01)** | **0.011** |
| Race |  |  |
| White | **Ref.** |  |
| African American | 0.96 (0.90-1.02) | 0.22 |
| Other | **0.75 (0.67-0.84)** | **<0.001** |
| Year of diagnosis |  |  |
| 2004 | **Ref.** |  |
| 2005 | 1.02 (0.90-1.16) | 0.78 |
| 2006 | 0.97 (0.84-1.09) | 0.49 |
| 2007 | 0.99 (0.87-1.12) | 0.86 |
| 2008 | 0.96 (0.85-1.09) | 0.55 |
| 2009 | 0.96 (0.85-1.09) | 0.55 |
| 2010 | 1.12 (0.93-1.34) | 0.24 |
| 2011 | 1.15 (0.96-1.38) | 0.13 |
| 2012 | 1.02 (0.84-1.22) | 0.87 |
| 2013 | 1.07 (0.89-1.28) | 0.5 |
| 2014 | 0.93 (0.77-1.12) | 0.44 |
| 2015 | 0.86 (0.71-1.03) | 0.11 |
| Gleason score |  |  |
| ≤6 | **Ref.** |  |
| 7 | **1.67 (1.12-2.47)** | **0.011** |
| ≥8 | **2.61 (1.78-3.81)** | **<0.001** |
| PSA |  |  |
| ≤20 | **Ref.** |  |
| ＞20 | **1.20 (1.10-1.31)** | **<0.001** |
| AJCC.T |  |  |
| T1-T2 | **Ref.** |  |
| T3-T4 | **1.21 (1.14-1.29)** | **<0.001** |
| AJCC.N |  |  |
| N0 | **Ref.** |  |
| N1 | **1.23 (1.16-1.31)** | **<0.001** |
| AJCC.M |  |  |
| M1a | **Ref.** |  |
| M1b | **1.63 (1.43-1.86)** | **<0.001** |
| M1c | **2.04 (1.77-2.34)** | **<0.001** |
| M1NOS | **1.79 (1.47-2.17)** | **<0.001** |
| CI = confidence interval; HR = hazard ratio; Ref. = reference; BR = beam radiation  AJCC = American Joint Committee on Cancer. | | |
